# Youth–Therapist and Parent–Therapist Match and Mismatch on Internalizing and Externalizing Treatment Goals as Predictors of Treatment Engagement

**DOI:** 10.3390/adolescents3040048

**Published:** 2023-12-13

**Authors:** May Yeh, Devynne Diaz, Argero Zerr, Alisandra Macias, Kristen McCabe

**Affiliations:** 1 Department of Psychology, San Diego State University, 5500 Campanile Dr., San Diego, CA 92182, USA; 2 Child and Adolescent Services Research Center, San Diego, CA 92123, USA; 3 Department of Psychiatry, University of California San Diego, 9500 Gilman Dr., La Jolla, CA 92093, USA; 4 Department of Psychology, California State University, Channel Islands, 1 University Dr., Camarillo, CA 93012, USA; 5 Department of Psychological Sciences, University of San Diego, 5998 Alcala Park, San Diego, CA 92110, USA

**Keywords:** treatment goals, client–therapist agreement, cultural competence, treatment engagement

## Abstract

Therapist–client cognitive match upon key constructs such as treatment goals is purported to be an important component of culturally competent care. For adolescent clients, treatment may involve both youths and their parents, suggesting the need to consider both youth–therapist and parent–therapist perspectives. This longitudinal study examined broadband youth–therapist and parent–therapist treatment goal matching and mismatching in relationship to treatment engagement in a culturally diverse sample of 245 outpatient mental health service-using youth. Although goal matching/mismatching did not uniformly predict treatment engagement as measured by a total score, youth–therapist internalizing goal matching predicted better youth engagement, and parent–therapist externalizing goal mismatch marginally predicted worse parent engagement. When selected post hoc analyses examined relationships to four individual engagement dimensions, youth–therapist internalizing goal matches positively predicted youth Client–therapist interaction, Communication/Openness, Client’s perceived usefulness of therapy, and Collaboration with treatment, while parent–therapist externalizing goal mismatch negatively predicted parent Collaboration with treatment. Findings support the importance of cognitive match on treatment goals as well as the consideration of both parent and youth perspectives, matched and mismatched goals, internalizing and externalizing goals, and examining individual dimensions in addition to total scores of engagement.

## 1. Introduction

It has been hypothesized that therapy for ethnic minority clients can be negatively affected by client–therapist cultural differences in treatment-related areas such as the conceptualization of the client’s problem, approaches to coping with or solving the problem, and treatment goals [1,2]. As such, therapist–client “cognitive match” on such factors is theorized to be an important element of culturally competent care [2]. Similarly, Kleinman and colleagues emphasize the need to understand and “negotiate” differences in patient and provider “explanatory models”, or culturally influenced explanations related to areas such as problem causes, problem severity, and/or aspects of treatment [3]. In addition, there is identification of the importance of “shared narratives” in culturally competent mental health care [4] and of finding what is “at stake” for clients [4,5]. Calls for the personalization of care also emphasize the need to tailor treatment to individuals [6,7], and a recent meta-analysis of personalized interventions found evidence that personalization can improve psychotherapy outcomes [8]. Others have identified the importance of client–therapist collaboration [9–12], a multiple informant approach when determining needs and treatment goals for youth [13], and the shared decision-making model (SDM, e.g., [14]) by which providers and consumers collaborate throughout care. In sum, there is recognition of the importance of shared perspectives, or matches between client and provider, upon key aspects of care.

Empirical support has been growing for the significance of shared perspectives between clients and providers in the provision of culturally competent care. In research with Asian American and White adults, Zane et al. [2] report that client–therapist cognitive match on treatment goals predicted session impact, matches on avoidant coping orientation resulted in less short-term dysphoria, and client–therapist similarities in perceived distress related to interpersonal problems predicted higher short-term psychosocial functioning. Youth–therapist co-endorsement of beliefs about the causes of the youth’s problems predicted youth treatment engagement in a culturally diverse sample of outpatient mental health service-using youth [15]. Further, “adaptation of the illness myth” was the only moderator of better outcomes in a meta-analysis of culturally adapted psychotherapy [16]. Thus, focusing on improving therapist–client cognitive match has the potential to increase the cultural sensitivity of mental health services.

When considering care for youth, it may be particularly meaningful to examine cognitive match in relationship to treatment engagement, given the evidence that about 50% of youths in treatment terminate services prematurely (e.g., [17,18]). In addition, the literature has indicated that Black and Hispanic youth have significantly lower mental health professional visit rates as compared to White children [19], and Black, Indigenous, and People of Color (BIPOC) have consistently lower treatment engagement in experimental trials of mental health treatment interventions as compared to non-Latino Whites [20], pointing to the importance of understanding factors that affect treatment engagement in culturally diverse populations. Moreover, Becker and Chorpita [18] cite the importance of treatment engagement in building the “bridge” that connects the “science” of efficacious intervention development with the “service” of therapists in community settings through “an intentional focus on the youths and families who participate in these interventions and who work with those therapists” (p. 284). In addition, identifying mechanisms that predict treatment engagement for a culturally diverse sample may help elucidate practices that are associated with culturally responsive care and may improve treatment retention and outcome.

Previous research on treatment engagement has examined engagement in general as well as by specific domain, and meaningful models characterize the dimensions of engagement [21]. For example, in the REACH organizing framework, Becker and colleagues have identified five domains of engagement: Relationship (e.g., therapist–client therapeutic alliance), Expectancy (e.g., perception of treatment helpfulness), Attendance, Clarity (e.g., understanding treatment approach and roles), and Homework (e.g., completion of homework, participation in sessions) [21]. There is some indication that therapist–client matches on important aspects of treatment, and goals in particular, may indeed be related to better treatment engagement in various ways. For example, Zane et al. [2] report in their research with Asian American and White adults that client–therapist cognitive matches on treatment goals predicted client ratings of session depth. In addition, youth-reported therapist understanding of one’s cultural background was shown to predict all five of the REACH dimensions of treatment engagement [22]. Moreover, in their examination of 50 randomized, controlled clinical trials of youth engagement interventions, Becker et al. [21] found that while some engagement strategies might be especially useful on an “as-needed” basis to facilitate a particular aspect of engagement, some practices, including that of goal setting (i.e., “Explicitly selecting a therapeutic goal for the purpose of making a plan toward achieving that goal”; p. 7), might be helpful in promoting engagement for all youth and their families. However, while engagement intervention studies involve intentional goal-setting practices, a related but separate question involves whether having actual youth–therapist or parent–therapist goal matches or mismatches may in and of itself have an impact on treatment engagement.

Treatment goal matching is an aspect of cognitive match that may be of particular significance to facilitating therapy processes and outcomes. Research suggests that psychotherapy outcomes such as retention, symptom reduction and adaptive functioning are improved when therapists and patients agree upon treatment goals in adult populations [23,24]. There is also some evidence for a relationship between youth–therapist treatment goal agreement and treatment outcomes in the context of youth treatment alliance. For example, in a sample of adolescent girls diagnosed with anorexia nervosa and their parents who received family-based therapy, achieving higher client–therapist (i.e., adolescent–therapist) goal agreement as a component of therapeutic alliance earlier in therapy was associated with adolescent weight gain [25]. Furthermore, discrepancies in client–therapist goal ratings may be related to poorer treatment outcomes. In a sample of adolescents who used substances, client–therapist (i.e., adolescent–therapist) goal agreement (*r* = 0.07, very small effect) and differences between client–therapist goal ratings (*r* = 0.38, medium effect) as part of therapeutic alliance were related to client participation in illegal activities [26]. These findings suggest that further examination of the effects of adolescent–therapist goal matching (and mismatching) in outpatient psychotherapy for youth more broadly may be warranted.

Since parents are often involved in initiating therapy and/or playing an ongoing role in treatment for youths, parents are an additional stakeholder to consider along with the youth and the therapist. Unfortunately, parent–youth–therapist agreement on treatment goals is poor [27,28]. For example, in one study, almost two-thirds of 170 parent-youth–therapist triads receiving community-based mental health services failed to agree on a single treatment goal [27]. In another sample of youth referred to community mental health centers and their parents, a similar lack of consensus on treatment goals was evident as 76.8% of the triads did not agree on a single specific target problem and less than half of 315 parent-youth–therapist triads agreed on one or more target problems at a more general problem type level [28]. Notably, treatment goal agreement was higher between parents and therapists than it was compared to youths and therapists when examining at least one specific target problem [28], suggesting the importance of examining parent–therapist and youth–therapist agreement separately.

Studies have also found that agreement on treatment goals between parent-youth–therapist triads is higher for externalizing problems as compared to internalizing problems [28]. These treatment goal findings parallel research on the presence of problem behaviors in youths as determined through symptomatology questionnaires, which indicates higher agreement between parent-youth dyads and interparental dyads on the presence of problems in the externalizing domain rather than the internalizing domain [29–31]. These similar results across agreement on treatment goals and agreement on behavior problem symptoms may be due to the more visible and objective quality of externalizing problems, such as stealing, property destruction, arguing, or defiant behavior, as compared to internalizing problems, such as sadness, social withdrawal, or anxiety. Therefore, when analyzing treatment goal matching, it may be important to consider treatment goals separately by broadband internalizing and externalizing problem types. In addition, as studies have indicated notably low levels of parent-youth–therapist agreement on specific goals for treatment, it may be useful to begin by examining whether more commonly observed agreement on a broader dimension, such as internalizing and externalizing, is sufficient to predict better engagement in therapy. Further, since symptomatology is often assessed and characterized as being either internalizing or externalizing in nature, a focus on cognitive matches upon these broadband domains is highly clinically relevant.

The present study investigated youth–therapist and parent–therapist treatment goal match/mismatch on broadband domains (internalizing or externalizing) in relationship to later treatment engagement in a culturally diverse sample of community outpatient service-using adolescents. This study enables further testing of the hypothesis that client–therapist matching on cognitive aspects of treatment, and specifically treatment goals, will be associated with clinically relevant outcomes, in this case, better treatment engagement, doing so in a youth population for which the relevance of adult theories cannot be assumed. The ability to examine cognitive match in relationship to treatment engagement in a culturally diverse sample is particularly important due to evidence of lower mental health visit rates for Black and Hispanic children [19] and lower engagement for BIPOC children in experimental intervention trials [20] as compared to Whites and due to the context of cognitive match hypotheses, whereby such client–therapist matches are viewed as a component of culturally competent care [2]. Both matches and mismatches will be examined, allowing for the study of the impact of goals that are shared in common as well as those that are identified by one individual but not the other. The examination of both youth–therapist and parent–therapist dyads enables consideration of the possibility that youth and parents may have differing thoughts about treatment goals, varying levels of engagement in treatment, and differential relationships between cognitive match and engagement. In addition, analyses will focus upon a total engagement score that involves interactional, communication, perceived utility, and collaborative aspects of engagement, but will also include selected post hoc analyses of these four specific engagement dimensions individually. Investigation of these relationships in a sample of youth receiving usual care in community settings can help identify mechanisms occurring in “usual care” or community settings that have ecological validity and the potential to inform efforts to improve care across both evidence-based practice and usual care in community settings (e.g., [27,28]). Finally, by looking at internalizing problem goals and externalizing problem goals separately, we are able to further understand if the effects of matching/mismatching on treatment engagement differs by broadband problem type.

## 2. Materials and Methods

### 2.1. Procedure

The study’s investigators recruited therapists delivering clinic-based and/or school-based outpatient psychotherapy to youth who were enrolled in a large, West Coast metropolitan school district in the United States, via letters, meetings, and/or telephone. Youth were eligible to be recruited if they were aged 12 and over, they were referred for clinic-based or school-based outpatient psychotherapy, they were students in the associated school district, and their therapist was participating in the study. Participating therapists presented study flyers to youths/parents and, following written permission for study contact, research staff phoned those families that expressed interest in the study. Families were excluded from the study if, during telephone screening, the parent/caregiver (hereafter called parent) reported youth receipt of a diagnosis of intellectual disability, pervasive developmental disorder, severe brain injury, or sensory impairment, or the parent reported that the youth was receiving specifically time-limited/involuntary counseling/psychotherapy, in foster care, involved in a special demonstration project, or using more intensive services in addition to the current outpatient psychotherapy. The study received Institutional Review Board approval. Therapist interviews took place in English, and parent/youth interviews were completed in English or Spanish. Longitudinal data collection took place across 5 potential timepoints covering approximately 1 year, involving a Time 1 interview and follow-up interviews targeted at 2 months, 4 months, 6 months, and 1 year after the Time 1 interview. Participants were given $30 in compensation/gift certificates for Time 1 assessments. Parents/youth were given $20 and therapists were given $10 in compensation/gift certificates for each follow-up assessment.

### 2.2. Measures

The present study utilized demographic information and treatment goal data collected at Time 1 via in-person interviews with youth, parents, and therapists, as well as treatment engagement data collected via telephone interviews with therapists at Time 2 (approximately 2 months after Time 1).

Demographic Information. Demographic information was obtained via structured inperson interviews. Parents were asked to provide their total household income by the week, month, or year, in whatever manner was easiest for them, and this was converted into a continuous annual household income variable. Parent highest level of schooling and receipt of additional certifications/training was obtained via interview, and parent education level for this study was categorized as follows: Up to grade 8, grades 9–12 (no diploma), high school/GED/additional certification, some college, college degree (and above).

#### Treatment Goals.

During separate interviews with research staff at Time 1, youths, parents, and therapists were each individually and independently asked an open-ended question about their treatment goals: “What are your goals for counseling?” (youths and therapists) or “What are your goals for your child’s counseling?” (parents). Codes were then assigned to responses based upon a system developed and utilized in previous research on target problem/treatment goal agreement [28,32]. The coding system uses items from the Child Behavior Checklist (CBCL; [33]) and Youth Self-Report (YSR; [34]) as the basis for codes that could then be placed into categories that parallel the narrowband syndromes (aggressive behavior, anxious/depressed, attention problems, delinquent behavior, sex problems, social problems, somatic complaints, thought problems, and withdrawn) and domains that included broadband syndromes (externalizing problems, internalizing problems). Codes were also developed for responses that were outside of the CBCL/YSR items, resulting in 3 additional non-narrowband categories: stressors, problems with daily living, and miscellaneous classification (e.g., child wants to acquire a skill, therapy mediator, such as developing the therapeutic relationship). Cohen’s kappa coefficients were computed to determine interrater agreement between coders on treatment goal categories outside of the miscellaneous category. Kappa coefficients for interrater agreement on the treatment goal categories ranged from 0.59 to 0.92 (average = 0.75) for the larger study, from which this subsample was drawn. Using Landis and Koch’s [35] classifications of agreement, the coders achieved moderate agreement on 1 category (stressors), substantial agreement on 5 categories (anxious/depressed, sex problems, social problems, thought problems, and withdrawn), and almost perfect agreement on 4 categories (aggressive behavior, attention problems, delinquent behavior, and problems with daily living). The current study will focus on goals in the broadband domains of internalizing problems (i.e., goals with codes in the anxious/depressed, somatic complaints, or withdrawn categories) and externalizing problems (i.e., goals with codes in the aggressive behavior, delinquent behavior categories).

##### Calculations of youth–therapist and parent–therapist matches and mismatches.

A match within pairs was determined in two ways for the present analyses: (1) An internalizing match (I-Match) occurred if both members of a pair named counseling goals that fell into the same broadband domain of internalizing problems. For example, if a youth were to report a goal related to the youth’s anxiety and the therapist were to indicate increasing the youth’s self-esteem as a goal, a broadband internalizing goal match would be present. (2) An externalizing match (E-Match) occurred if both members of a pair indicated therapy goals that were in the same broadband domain of externalizing problems.

Mismatch within a pair was determined in two ways: (1) An internalizing mismatch (I-Mismatch) occurred if one person voiced a treatment goal that fell into the internalizing domain, but the other did not mention any treatment goals in that domain. For example, if the youth mentioned counseling goals related to anxiety (an internalizing goal) and also fighting with peers (an externalizing goal), but all of the therapist’s goals related to externalizing issues, then a youth–therapist internalizing mismatch would be present. (2) Similarly, an externalizing mismatch (E-Mismatch) occurred if one person mentioned a goal that fell into the externalizing domain, but the other did not voice any counseling goals in that domain.

#### Treatment Engagement.

The engagement measure [36] involves six therapy engagement dimensions assessed through 11 therapist reported items that are rated on a 5-point Likert scale (1–5, with higher scores indicating better engagement) for Appointment keeping (2 items), Client–therapist interaction (1 item), Communication/Openness (3 items), Client’s perceived usefulness of treatment (1 item), Collaboration with treatment (3 items), and Compliance with medication (1 item). Good face and discriminatory validity, good test–retest reliability (item correlations = 0.71–0.84; total score correlation = 0.90), and good inter-rater reliability (item correlations = 0.86–1.0; total score correlation = 0.95) have previously been reported for the measure [36]. Ratings across the 11 items are summed to create a total engagement score for the original measure. The measure was adapted for the current study to allow for therapist ratings of youth and parent engagement separately in treatment as well as to enable telephone administration. For example, references to the “client” were replaced with “youth” or “youth’s caregiver” when appropriate, and topic headings (e.g., “Client’s perceived usefulness of treatment”) with ratings designed for paper/written administration were reworded into topical questions (e.g., “How often does the youth’s caregiver perceive the treatment to be useful?”) with ratings that were more easily understood when administered verbally. Due to circumstances where items may not be relevant (e.g., if the youth always received support for attending treatment and the item inquired about youth attendance of treatment without support), “Not Applicable” responses were allowed and treated as missing values. The medication compliance item was omitted from the total engagement sum score for the present study due to concerns about the validity of ratings from therapists who would not have been the medication prescribers. Following careful consideration, the two appointment keeping items (attendance without support, attendance with support) were also omitted due to concern of lack of applicability of one or the other of the items for some of our participants (for example, it is possible that some youth attending treatment in school settings may have always had support for attendance). The resulting eight-item total engagement sum score ranged from 8 to 40 (higher scores indicate better engagement) that involves four dimensions of engagement: Client–therapist interaction, Communication/Openness, Client’s perceived usefulness of treatment, Collaboration with treatment. These can be conceptualized as aligning with the Relationship, Expectancy, and Homework domains of the REACH framework [21]. Others have also used the measure in a shortened, continuous form with adolescents (e.g., as a 9-item, continuous scale with adolescent sexual offenders [37]). The modified measure was administered via telephone to therapists at the Time 2 interview, which was targeted for approximately 2 months after the Time 1 interview, enabling examination of Time 1 treatment goal agreement in relationship to engagement at the study’s earliest longitudinal engagement timepoint.

### 2.3. Participants

Youth, parent, and therapist participants were involved in a longitudinal study investigating youth/parent/therapist cognitive consensus, or cognitive matching, on multiple treatment-related constructs for 318 youth who were receiving clinic-based or school-based outpatient therapy. In order to focus upon agreement at earlier stages of treatment, cases were excluded from the present analyses if the youth/parent interviews occurred more than 30 days after the initial treatment session and therapist interviews occurred more than 5 sessions after the initial treatment session. Exclusions also occurred due to clustering considerations (a clinic with a single study case, two siblings who both participated in the study). These design-related exclusions resulted in a sample of 285. Then, of this sample of 285, cases were retained if they had either complete youth data (*n* = 231) or complete parent data (*n* = 206) for the variables included in the focal analyses, resulting in data associated with 245 youth. Comparisons between those who were selected for the analyses (*n* = 245) and those who were not selected from the original sample (*n* = 73) yielded a significant difference in household income (*t* [145.43] = 2.57, *p* = 0.01; higher for the selected sample) but no differences in Time 1 youth age, youth gender, or the proportion of the sample that was Latinx.

Youths associated with this selected sample (*n* = 245) were aged 12–18 years (*M* = 14.09; *SD* = 1.56) at the Time 1 interview, and 59.2% were male while 40.8% were female. Of the youth, 70.6% were Latino/Hispanic/Spanish of any race (referred to hereafter as Latinx). In addition to the youth that were Latinx, 12.7% were African American/Black, 0.4% were American Indian/Native American/Alaska Native, 2.4% were Asian American/Pacific Islander, 6.9% were Multiracial, 5.7% were non-Hispanic White, and 1.2% were missing this information. The youth received school-based services (81.2%) or clinic-based services (18.8%).

Parents associated with this selected sample (*n* = 245) had an average age of 41.77 (*SD* = 8.65; missing *n* = 8) at the Time 1 interview, and 89.4% were female while 10.6% were male. Of the parents, 68.2% were Latinx. In addition to those parents who were Latinx, 14.3% were African American/Black, 0.4% were American Indian/Native American/Alaska Native, 2.4% were Asian American/Pacific Islander, 4.9% were Multiracial, 9.0% were non-Hispanic White, and 0.8% were missing this information. Mean household income was $22,722 (*SD* = $14,728; 2.9% missing). The highest educational levels of parents were: 27.8% through 8th grade, 14.3% grades 9–12 (no diploma), 25.7% high school diploma, GED, or additional certification, 21.6% some college education but no degree, 9.8% a college degree or above, and 0.8% (*n* = 2) were missing education information.

In the therapist sample (*n* = 46), 87% were female, and 13% were male. Of the therapists, 41.3% were Latinx. In addition to those who were Latinx, 4.3% were African American/Black, 6.5% were Asian American/Pacific Islander, and 47.8% were non-Hispanic White. At the time of the therapist’s first study interview, the therapists’ highest educational levels were: bachelor’s degree (4.3%) or master’s degree (95.7%).

### 2.4. Data Analytic Plan

All models controlled for the following service-related variables: (1) location of service provision (school-based versus clinic-based); (2) multisystemic therapy (MST) versus not, to account for possible differences between MST and traditional outpatient services; and (3) the number of sessions prior to the therapist Time 1 interview. In addition, correlational and t-test analyses were conducted to determine demographic variables to include as covariates. Primary and post hoc analyses utilized mixed effects modeling to take therapist clustering into account as a random effect, using the restricted maximum likelihood (REML) method of estimation, and the variance components (VC) covariance structure. Data analyses used IBM SPSS Statistics for Windows, Version 29 [38].

1. *Broadband Goal Match:* We first examined if broadband treatment goal matching (internalizing goal/I-Match; externalizing goal/E-Match) predicted Time 2 therapist-rated treatment engagement for youths and parents. The following four models were tested:
  - Does youth–therapist matching on an internalizing goal (YT I-Match) predict Time 2 youth engagement?
  - Does youth–therapist matching on an externalizing goal (YT E-Match) predict Time 2 youth engagement?
  - Does parent–therapist matching on an internalizing goal (PT I-Match) predict Time 2 parent engagement?
  - Does parent–therapist matching on an externalizing goal (PT E-Match) predict Time 2 parent engagement?
2. *Broadband Goal Mismatch:* We then examined if broadband treatment goal mismatching (internalizing goal/I-Mismatch; externalizing goal/E-Mismatch) predicted Time 2 therapist-rated treatment engagement. The following four models were tested:
  - Does youth–therapist mismatching on an internalizing goal (YT I-Mismatch) predict Time 2 youth engagement?
  - Does youth–therapist mismatching on an externalizing goal (YT E-Mismatch) predict Time 2 youth engagement?
  - Does parent–therapist mismatching on an internalizing goal (PT I-Mismatch) predict Time 2 parent engagement?
  - Does parent–therapist mismatching on an externalizing goal (PT E-Mismatch) predict Time 2 parent engagement?

## 3. Results

### 3.1. Preliminary Analyses

Preliminary analyses were conducted for Time 1 youth demographic variables (youth age, youth gender) in relationship to Time 2 youth engagement, and Time 1 parent demographics (parent age, annual household income, parent highest level of education) in relationship to Time 2 parent engagement (*n* = 245). A significant positive correlation was found between youth age and youth treatment engagement (*r* = 0.16, *p* = 0.02), and females were found to have significantly higher treatment engagement compared to males (*t* [232] = 4.50, *p* < 0.001); therefore, youth age and gender were included as control variables in analyses where youth engagement was a dependent variable. A significant negative correlation was found between annual household income and parent treatment engagement (*r* = −0.16, *p* = 0.02), and parent education level was significantly negatively associated with parent treatment engagement (Spearman’s *ρ* = −0.14, *p* = 0.04); therefore, household income and parent education level were used as control variables in analyses for which parent engagement was the dependent variable.

### 3.2. Question 1: Broadband Treatment Goal Match

The first set of models examined the relationship between treatment goal matching (as defined by both persons voicing a problem that fell into the same broadband domain) as it related to treatment engagement at Time 2, while controlling for relevant demographic and service-related variables. Internalizing and externalizing treatment goal matching were examined separately. Internalizing matches occurred in 15.6% (*n* = 36) of youth–therapist dyads and 24.3% (*n* = 50) parent–therapist dyads, while externalizing matches occurred in 35.9% (*n* = 83) of youth–therapist dyads and 38.8% (*n* = 80) parent–therapist dyads. Youth–therapist I-Match was significantly related to overall youth treatment engagement (*B* = 3.33, *p* = 0.002), meaning that when youth and therapists both voiced a treatment goal in the internalizing domain, this predicted better youth treatment engagement approximately two months later. However, youth–therapist E-Match was not significantly related to total treatment engagement for youths (*B* = 0.31, *p* = 0.68). Parent–therapist I-Match was not significantly related to Time 2 parent engagement (*B* = −0.92, *p* = 0.30), nor was parent–therapist E-Match (*B* = 1.09, *p* = 0.16). See Table 1.

### 3.3. Question 2: Broadband Treatment Goal Mismatch

The second set of models examined the relationship between treatment goal mismatching (as defined by one person voicing a problem in one domain when the other did not) and treatment engagement at Time 2, while controlling for relevant demographic and service-related variables. Internalizing and externalizing treatment goal mismatching were examined separately.

I-Mismatches occurred for 39.8% (*n* = 92) of youth–therapist dyads, with 12.0% (*n* = 11) of these involving cases where only the youth mentioned an internalizing goal while the therapist did not, and 88.0% (*n* = 81) of cases where only the therapist mentioned an internalizing goal while the youth did not. I-Mismatches were present for 37.9% (*n* = 78) of parent–therapist pairs, with 32.1% (*n* = 25) of these mismatches involving cases where only the parent mentioned an internalizing goal while the therapist did not, and 67.9% (*n* = 53) representing cases where only the therapist mentioned an internalizing goal, but the parent did not.

E-Mismatches occurred for 44.2% (*n* = 102) of youth–therapist dyads, with 10.8% (*n* = 11) of these involving cases where only the youth mentioned an externalizing goal while the therapist did not, and 89.2% (*n* = 91) of cases where only the therapist mentioned an externalizing goal while the youth did not. E-Mismatches were present for 48.1% (*n* = 99) of parent–therapist pairs, with 22.2% (*n* = 22) of these mismatches involving cases where only the parent mentioned an externalizing goal while the therapist did not and 77.8% (*n* = 77) representing cases where only the therapist mentioned an externalizing goal but the parent did not.

Youth–therapist I-Mismatch was not significantly related to total youth treatment engagement (*B* = −1.09, *p* = 0.15), nor was youth–therapist E-Mismatch (*B* = −0.74, *p* = 0.31). Parent–therapist I-Mismatch was also not significantly related to overall parent treatment engagement (*B* = 0.51, *p* = 0.49), but parent–therapist E-Mismatch was marginally related to parent treatment engagement (*B* = −1.40, *p* = 0.055). This indicated that when either the parent or the therapist, but not both, voiced an externalizing treatment goal, this mismatch marginally predicted worse parent treatment engagement approximately two months later. See Table 2.

### 3.4. Post Hoc Analyses

Post hoc analyses were conducted to examine whether the relationship between youth–therapist I-Match was consistent across four engagement dimensions while controlling for Time 1 youth age, youth gender, and service-related variables. Youth–therapist I-Match was a positive predictor of Time 2 Client–therapist interaction (*B* = 0.33, *p* = 0.03), Communication/Openness (*B* = 1.42, *p* = 0.003), Client’s perceived usefulness of therapy (*B* = 0.57, *p* < 0.001), and Collaboration with treatment (*B* = 1.01, *p* = 0.04).

Following careful consideration, parallel post hoc analyses were also conducted to investigate whether the marginally significant relationship between parent–therapist E-Mismatch was consistent across four engagement dimensions, also while controlling for Time 1 household income and service-related variables. This decision was made due to the possibility that our total engagement score obscured important relationships between cognitive match and the four dimensions of engagement in our total engagement variable and also due to the potential clinical significance of parent–therapist mismatch on externalizing problem goals, which are a frequent presenting problem in treatment. Parent–therapist E-Mismatch was a significant negative predictor of Time 2 Collaboration with treatment (*B* = *−*0.68, *p* = 0.045), but not of the other three dimensions of Client–therapist interaction (*B* = *−*0.14, *p* = 0.21), Communication/Openness (*B* = *−*0.39, *p* = 0.20), and Perceived usefulness of treatment (*B* = *−*0.13, *p* = 0.25).

## 4. Discussion

This study investigated treatment goal matching and mismatching on internalizing and externalizing treatment goals as predictors of treatment engagement in a culturally diverse sample of youth receiving community-based outpatient therapy. Some evidence was found for the role of goal matching in predicting treatment engagement and for mismatching to predict worse treatment engagement, and as such, support emerged for cognitive match [1,2] as it relates to treatment goal matching in youths, but only under certain conditions. The findings point to the importance of examining whether clients match or mismatch with therapists on treatment goals, doing so with attention to both youth and parent perspectives, internalizing and externalizing goals, and total and dimensional scores of engagement.

First, we examined whether matching on broadband internalizing/externalizing treatment goals predicted later treatment engagement as defined by a total score involving Client–therapist interaction, Communication/ Openness, Client’s perceived usefulness of treatment, and Collaboration with treatment items of the engagement measure [36] that were adapted for this study. We found that broadband goal match predicted later treatment engagement, but only for youth–therapist pairs where both persons voiced an internalizing treatment goal, but not for youth–therapist pairs where both persons voiced an externalizing goal. It is possible that these findings result from differences in youth perceptions regarding the utility of therapy for different types of problems; perhaps youths see therapy as more helpful for internalizing than externalizing problems. Or, due to the less visible nature of internalizing problems, it is possible that matching on these types of goals is more indicative of a stronger working alliance than is matching on externalizing goals that may be more obvious to observers. The level of motivation for youths to engage in treatment may also differ for internalizing as compared with externalizing issues. In addition, it may be particularly important for youths who come from cultures where mental health problems are especially stigmatized to have a place to discuss internalizing issues such as depression and anxiety. Notably, parent–therapist matching was not related to parent engagement for either internalizing or externalizing goals, indicating the utility of examining parent and youth engagement separately. In post hoc analyses of effects on four dimensions of treatment engagement, youth–therapist internalizing treatment goal matching was a predictor of Client–therapist interaction, Communication/Openness, Client’s perceived usefulness of therapy, and Collaboration with treatment. These findings suggest that internalizing goal matching seems to have an effect on multiple aspects of the working relationship between the youth and the therapist.

Second, when examining the predictive effects of mismatching, we did not find significant effects for parent–therapist internalizing mismatches or either of the youth–therapist mismatches, but mismatches for parent–therapist pairs were marginally predictive of worse treatment engagement. This is concerning, as a parent–therapist mismatch on externalizing goals was present in almost half (48.1%) of parent–therapist dyads. It is interesting to note that when parent–therapist pairs were mismatched on externalizing goals, less than a quarter (22.2%) of these were instances where the parent mentioned an externalizing problem while the therapist did not, while over three quarters (77.8%) of the time, it was the therapist who voiced an externalizing goal while the parent did not. Similarly, with parent–therapist internalizing mismatches, about one third (32.1%) of these were goals voiced only by the parent while about two-thirds (67.9%) were voiced only by the therapist. The picture emerges of therapists who are raising more externalizing and internalizing treatment goals than are parents and of less parental engagement when parents do not have externalizing treatment goals that therapists hold. Post hoc analyses of four engagement dimensions as outcome variables revealed that parent–therapist externalizing mismatch was specifically predictive of therapist ratings of the parent’s Collaboration with treatment, further pointing to the impact of such a mismatch on therapist perceptions of parent cooperation with treatment. In addition, these results highlight the possibility that goal matching may have a variable effect on different dimensions of engagement and point to the utility of examining individual domains of engagement (e.g., from the REACH framework, [21]) separately.

Taken as a whole, the findings provide some evidence that youth–therapist and parent–therapist cognitive matching on treatment goals may increase treatment engagement in a culturally diverse sample of youth, thus supporting the relevance of cognitive match in the promotion of culturally competent care for youth populations. Notably, the effects were found from data collected during individual research interviews (without the other member of the dyad present) that may have reflected more genuine responses than those given in a treatment setting driven by the therapist; this potentially underlines the importance of truly collaborative goal setting, and not simply goal setting whereby therapists dictate an agenda. Future research may be able to examine the relative contributions of matched goals (in and of themselves) and intentional goal-setting activities (that may or may not result in matched goals). Relatedly, it may be important to understand if therapist-initiated goal setting results in stronger goal matches, if this is moderated by degree of collaboration, and if there are changes in youth, parent, or therapist goals over the course of discussions.

These findings also support the need to consider various characteristics of treatment goal matching and mismatching when examining associated effects. Indeed, while research findings from Zane et al. [2] support the importance of cognitive matching, the authors indicate the absence of a “halo effect”, wherein matching of every kind led to uniform session impact and outcomes. Our findings also support the importance of specification, highlighting the need to examine youth as well as parent perspectives, internalizing and externalizing treatment goals, and individual dimensions of engagement in addition to total scores. Each informant may provide pertinent information across various domains that may need to be integrated when formulating treatment goals [13,39]. As such, future studies should also examine similar issues in parent–youth dyads. Investigating other areas of engagement such as appointment keeping and also engagement at both earlier and later timepoints may give a greater sense of how and when treatment goal matching and mismatching may be most impactful.

The study findings should be interpreted in light of several limitations. First, we focused on matching and mismatching as determined by broadband domains of symptomatology. This method allowed us to understand if goal matching or mismatching on an internalizing or externalizing broadband level would affect treatment engagement. However, looking at matching and mismatching in other ways, such as the specific type of goal (e.g., depression versus anxiety) may have yielded different findings. Second, the measure of treatment engagement in this study was based upon therapist reports, and we adapted the measure to allow for telephone administration and separate ratings of parent and youth engagement. Behavioral observations, youth reporting, or parent reporting of treatment engagement may have produced different findings. Further, we omitted appointment-keeping items from the original measure, due to concerns about the relevance of these items to our sample (e.g., those that were receiving school-based services with support), but this meant that appointment keeping, which is an often-studied aspect of engagement, was not represented in our study. Third, we interpreted a marginally significant finding (*p* = 0.055), due to the clinical meaningfulness of the results and the possibility that our total treatment engagement score may have obscured differential effects for the multiple dimensions of engagement, but this does not conform with typical norms of a cut-off of *p* < 0.05 for statistical significance. Thus, this finding, and the post hoc analyses that followed from it, must be interpreted with caution. However, the post hoc findings also support the utility of examining individual dimensions of engagement in addition to total scores. Fourth, the study had the strength of involving naturally occurring outpatient therapy and thus greater ecological validity, but this meant that treatment standardization did not occur. Although the analyses took therapist clustering into account, receipt of varying levels of psychoeducation regarding treatment and different types of therapy may have affected how youth and parents defined their treatment goals. Fifth, clients may have had varying levels of exposure to previous mental health care that may have impacted the way in which they identified treatment goals, and we did not take this variable into account as a potential moderator in our analyses. We also did not take the severity of mental health issues or family dynamics into account. Sixth, we treated “not applicable” responses as missing values and not as zero values with the engagement measure. This may have resulted in a greater loss of participants from our analyses. Finally, we focused on treatment goals that reflected internalizing/externalizing symptomatology; future research may examine the effects of matching/mismatching on counseling goals that may be outside of these broadband areas or that may not be classified as symptoms.

## 5. Conclusions

Overall, the findings suggest that matching or mismatching on treatment goals affected the treatment engagement for our culturally diverse sample under certain conditions. Specifically, youth–therapist matching on internalizing treatment goals and parent–therapist mismatching on externalizing goals may be particularly impactful for treatment engagement. Future research should examine ways to improve youth–therapist match on internalizing treatment goals and parent–therapist match on externalizing goals. Our data suggest that therapists may be voicing more internalizing and externalizing treatment goals than either parents or youths, such that therapists may have the opportunity to increase treatment engagement through ensuring that youths and parents are aware of and supportive of their treatment goals. Intentional elicitation of youth and parent explanatory models, as well as efforts to reconcile these with therapist explanatory models [3,5], is recommended for facilitating the process of developing culturally responsive, mutually agreed upon treatment goals that reflect client perspectives and “what is at stake” for clients [4,5]. In addition, shared decision making may be explored as a means for developing consensus upon treatment goals [40]. Moreover, it would be important to examine treatment goal matching and mismatching as potential predictors of other treatment-related outcomes such as symptomatology, functional impairment, and client satisfaction with treatment.

## Figures and Tables

**Table 1.** Youth–Therapist and Parent–Therapist Match on Internalizing and Externalizing Treatment Goals as Predictors of Youth and Parent Engagement.

	Total Youth Engagement (*n* = 231)		
Model	*B*	*SE*	95% CI
Youth–Therapist Match on Internalizing Goal			
Intercept	15.78 ***	3.85	[8.19, 23.36]
Youth gender	3.64 ***	0.73	[2.20, 5.08]
Youth age	0.37	0.26	[−0.15, 87]
Service location	1.02	1.45	[−1.86, 3.90]
Multisystemic therapy	−4.06 ^†^	2.06	[−8.16, −0.03]
Number of sessions	0.64 **	0.23	[0.18, 1.10]
YT match on internalizing goal	3.33 **	1.08	[1.20, 5.47]
Youth–Therapist Match on Externalizing Goal			
Intercept	13.91 ***	3.95	[6.12, 21.69]
Youth gender	3.65 ***	0.75	[2.18, 5.12]
Youth age	0.53 *	0.26	[0.01, 1.05]
Service location	1.63	1.48	[−1.30, 4.56]
Multisystemic therapy	−4.32 *	2.12	[−8.53, −0.12]
Number of sessions	0.66 **	0.24	[0.19, 1.13]
YT match on externalizing goal	0.31	0.75	[−1.16, 1.78]
	**Total Parent Engagement (*n* = 206)**		
**Model**	***B***	***SE***	**95% CI**
Parent–Therapist Match on Internalizing Goal			
Intercept	30.04 ***	1.36	[33.95, 40.32]
Household income	−5.65 × 10^−5^ *	2.74 × 10^−5^	[−0.00, −2.38 × 10^−6^]
Parent education level	−0.21	0.28	[−0.77, 0.36]
Service location	−0.32	1.36	[−3.02, 2.39]
Multisystemic therapy	4.13 *	1.78	[0.54, 7.72]
Number of sessions	0.54 *	0.23	[0.09, 0.99]
PT match on internalizing goal	−0.92	0.88	[−2.66, 0.81]
Parent–Therapist Match on Externalizing Goal			
Intercept	29.64 ***	1.38	[26.92, 32.37]
Household income	−6.15 × 10^−5^ *	2.69 × 10^−5^	[−0.00, −8.52 × 10^−6^]
Parent education level	−0.26	0.28	[−0.81, 0.30]
Service location	−0.41	1.37	[−3.13, 2.32]
Multisystemic therapy	3.81 *	1.83	[0.13, 7.49]
Number of sessions	0.55 *	0.23	[0.10, 0.99]
PT match on externalizing goal	1.09	0.77	[−0.42, 2.60]

*Note.* Youth gender: 1 = male, 2 = female; service location: 0 = school-based, 1 = clinic-based services; number of sessions = number of sessions before therapist Time 1 interview; CI = confidence interval.

† *p* < 0.10

* *p* < 0.05

** *p* < 0.01

*** *p* < 0.001.

**Table 2.** Youth–Therapist and Parent–Therapist Mismatch on Internalizing and Externalizing Treatment Goals as Predictors of Youth and Parent Engagement.

	Total Youth Engagement (*n* = 231)		
Model	*B*	*SE*	95% CI
Youth–Therapist Mismatch on Internalizing Goal			
Intercept	14.61 ***	3.89	[6.93, 22.28]
Youth gender	3.78 ***	0.75	[2.31, 5.25]
Youth age	0.51 ^†^	0.26	[−0.01, 1.03]
Service location	1.52	1.48	[−1.41, 4.46]
Multisystemic therapy	−4.23 *	2.12	[−8.45, −0.02]
Number of sessions	0.64 **	0.24	[0.18, 1.11]
YT mismatch on internalizing goal	−1.09	0.75	[−2.57, 0.38]
Youth–Therapist Mismatch on Externalizing Goal			
Intercept	14.31 ***	3.89	[6.64, 21.98]
Youth gender	3.64 ***	0.75	[2.18, 5.11]
Youth age	0.54 *	0.26	[0.02, 1.06]
Service location	1.57	1.48	[−1.36, 4.50]
Multisystemic therapy	−4.35 *	2.11	[−8.54, −0.15]
Number of sessions	0.64 **	0.24	[0.17, 1.11]
YT mismatch on externalizing goal	−0.74	0.72	[−2.15, 0.68]
	**Total Parent Engagement (*n* = 206)**		
**Model**	***B***	***SE***	**95% CI**
Parent–Therapist Mismatch on Internalizing Goal			
Intercept	29.73 ***	1.41	[26.94, 32.53]
Household income	−5.92 × 10^−5^ *	2.73 × 10^−5^	[−0.00, −5.45 × 10^−6^]
Parent education level	−0.24	0.28	[−0.80, 0.32]
Service location	−0.35	1.36	[−3.06, 2.36]
Multisystemic therapy	4.40 *	1.77	[0.84, 7.97]
Number of sessions	0.54 *	0.23	[0.09, 0.99]
PT mismatch on internalizing goal	0.51	0.74	[−0.96, 1.99]
Parent–Therapist Mismatch on Externalizing Goal			
Intercept	30.78 ***	1.43	[27.96, 33.60]
Household income	−5.84 × 10^−5^ *	2.68 × 10^−5^	[−0.00, −5.50 × 10^−6^]
Parent education level	−0.28	0.28	[−0.98, 0.28]
Service location	−0.52 *	1.36	[−3.23, 2.20]
Multisystemic therapy	3.75 *	1.80	[0.11, 7.38]
Number of sessions	0.54 *	0.23	[0.09, 0.98]
PT mismatch on externalizing goal	−1.40 ^†^	0.72	[−2.82, 0.03]

*Note.* Youth gender: 1 = male, 2 = female; service location: 0 = school-based, 1 = clinic-based services; number of sessions = number of sessions before therapist Time 1 interview; CI = confidence interval.

† *p* < 0.10

* *p* < 0.05

** *p* < 0.01

*** *p* < 0.001.

## Data Availability

Data presented in this study are not available for sharing.
